# Direct inhibition of STAT signaling by platinum drugs contributes to their anti-cancer activity

**DOI:** 10.18632/oncotarget.17661

**Published:** 2017-05-07

**Authors:** Stanleyson V. Hato, Carl G. Figdor, Susumu Takahashi, Anja E. Pen, Altuna Halilovic, Kalijn F. Bol, Angela Vasaturo, Yukie Inoue, Nienke de Haas, Dagmar Verweij, Carla M.L. Van Herpen, Johannes H. Kaanders, Johan H.J.M. van Krieken, Hanneke W.M. Van Laarhoven, Gerrit K.J. Hooijer, Cornelis J.A. Punt, Akira Asai, I. Jolanda M. de Vries, W.J. Lesterhuis

**Affiliations:** ^1^ Department of Tumor Immunology, Radboud University Medical Centre and Radboud Institute for Molecular Life Sciences, Nijmegen, The Netherlands; ^2^ Department of Pathology, Radboud University Medical Centre and Radboud Institute for Molecular Life Sciences, Nijmegen, The Netherlands; ^3^ Department of Medical Oncology, Radboud University Medical Centre and Radboud Institute for Molecular Life Sciences, Nijmegen, The Netherlands; ^4^ Department of Radiation Oncology, Radboud University Medical Centre and Radboud Institute for Molecular Life Sciences, Nijmegen, The Netherlands; ^5^ Center for Drug Discovery, Graduate School of Pharmaceutical Sciences, University of Shizuoka, Shizuoka, Japan; ^6^ Department of Medical Oncology, Academic Medical Centre, University of Amsterdam, Amsterdam, The Netherlands; ^7^ Current address: University of Western Australia, School of Medicine and Pharmacology, Perth, Australia

**Keywords:** platinum chemotherapy, STAT signaling, SH2 domain, cancer, STAT3

## Abstract

Platinum-based chemotherapeutics are amongst the most powerful anti-cancer drugs. Although their exact mechanism of action is not well understood, it is thought to be mediated through covalent DNA binding. We investigated the effect of platinum-based chemotherapeutics on signaling through signal transducer and activator of transcription (STAT) proteins, which are involved in many oncogenic signaling pathways.

We performed *in vitro* experiments in various cancer cell lines, investigating the effects of platinum chemotherapeutics on STAT phosphorylation and nuclear translocation, the expression of STAT-modulating proteins and downstream signaling pathways. Direct binding of platinum to STAT proteins was assessed using an AlphaScreen assay. Nuclear STAT3 expression was determined by immunohistochemistry and correlated with disease-free survival in retrospective cohorts of head and neck squamous cell carcinoma (HNSCC) patients treated with cisplatin-based chemoradiotherapy (*n* *=* 65) or with radiotherapy alone (*n* = 32).

At clinically relevant concentrations, platinum compounds inhibited STAT phosphorylation, resulting in loss of constitutively activated STAT proteins in multiple distinct cancer cell lines. Platinum drugs specifically inhibited phospho-tyrosine binding to SH2 domains, thereby blocking STAT activation, and subsequently downregulating pro-survival- and anti-apoptotic- target genes. Importantly, we found that active STAT3 in tumors directly correlated with response to cisplatin-based chemoradiotherapy in HNSCC patients (*p* = 0.006).

These findings provide insight into a novel, non-DNA-targeted mechanism of action of platinum drugs, and could be leveraged into the use of STAT expression as predictive biomarker for cisplatin chemotherapy and to potentiate other therapeutic strategies such as immunotherapy.

## INTRODUCTION

Platinum-based chemotherapeutic compounds are widely used in the treatment of a variety of human malignancies. These compounds enter the cell and undergo hydrolysis, giving rise to highly reactive aqua derivatives that bind to nucleophilic groups containing oxygen, nitrogen or sulfur donors [1]. Although these groups are ubiquitous in cells, in proteins, RNA and DNA, the covalent binding to DNA is widely accepted as the main mechanism of action [2]. However, of the covalently bound platinum in a cell, only 5–10% is bound to DNA, leaving the majority of the compound that enters the cell ending up as a protein/platinum complex [3]. Importantly, not only have several cellular proteins and RNA been found to bind to platinum drugs, but some of these interactions also resulted in conformational changes and altered biological function [4]. Non-DNA binding effects of platinum drugs, in particular on cellular signaling pathways, are still poorly understood [4].

One pathway of particular importance for platinum effects on cancer signaling is the signal transducer and activator of transcription (STAT) pathway. We recently showed that platinum drugs inhibit STAT6 phosphorylation in dendritic cells and a melanoma cell line [5], and others have found that non-clinically used platinum compounds can interfere with STAT3 binding to DNA [6, 7]. STAT signaling is initiated through ligand recognition by cytokine receptors, resulting in downstream Janus protein tyrosine kinase (JAK)-mediated phosphorylation of the SRC homology 2 (SH2) domain of STAT proteins. After phosphorylation-induced STAT homo- or heterodimerization, the STAT dimers translocate to the nucleus where they regulate the transcription of STAT target genes. Constitutive STAT activation is found in many tumors and plays an important role in cancer initiation, progression and immune escape [8, 9]. Due to their oncogenic potential STAT3, STAT5, and STAT6 are attractive targets for therapeutic intervention and STAT inhibitors are in development as anti-cancer drugs [8]. Here, we investigated the capability of platinum-based chemotherapeutics to directly interfere with STAT signaling proteins, and we assessed the clinical relevance of this interaction in cancer patients.

## RESULTS

### Platinum compounds directly inhibit STAT protein phosphorylation

**Figure 1:**
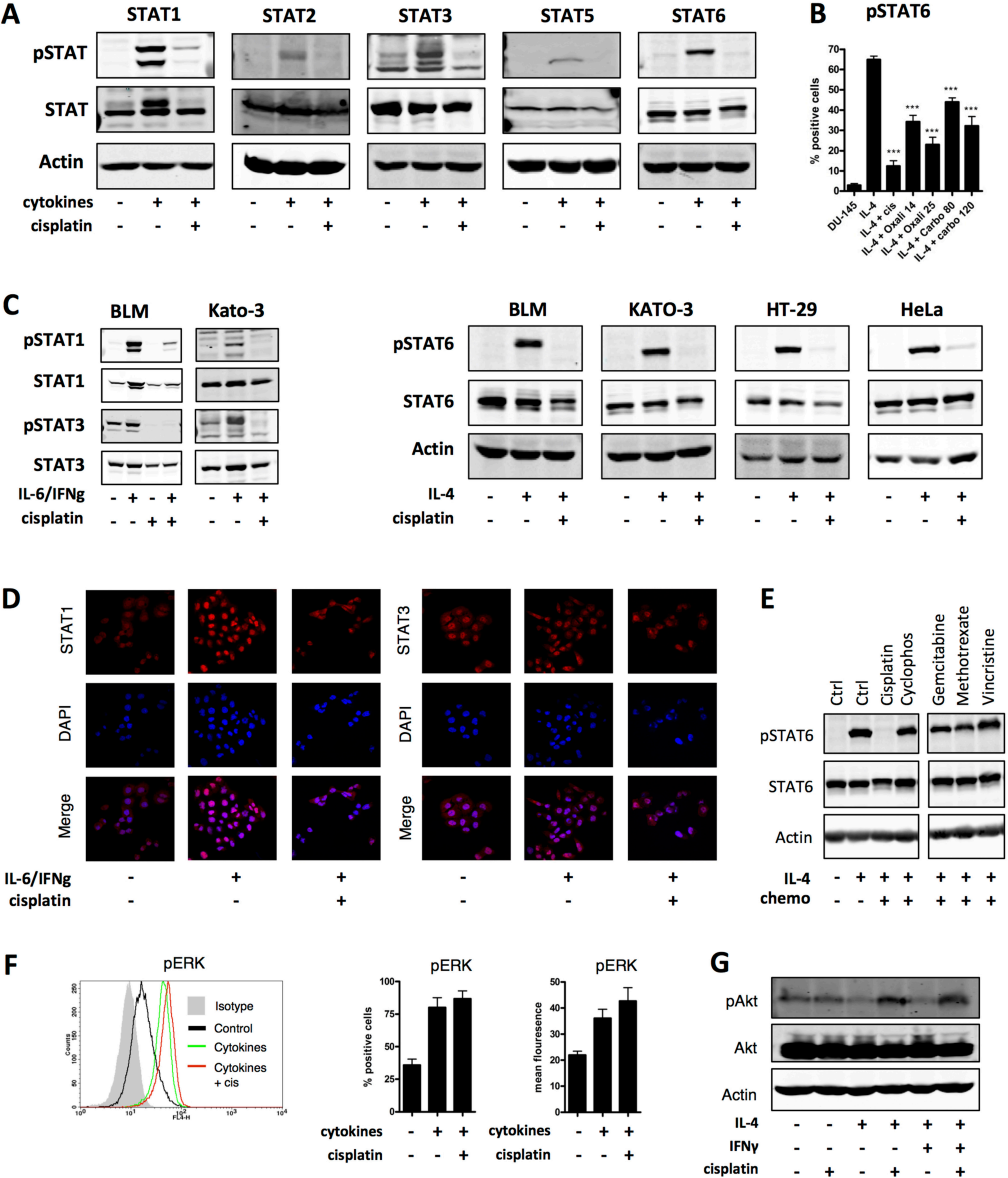
Platinum drugs specifically inhibit phosphorylation of STATs in multiple cancers (**A**) DU-145 cells were treated with the following cytokines: IL-6/IFNγ (STAT1 and STAT3), IFNα (STAT2), IL-4/IL-13/IFNγ (STAT5) or IL-4 (STAT6), to induce STAT protein phosphorylation, with and without simultaneous co-administration of cisplatin (10 μg/ml) for 18 hours. STAT protein expression and phosphorylation was analyzed by western blot. Actin is shown as a loading control. Shown is one representative experiment out of at least four independent experiments. (**B**) DU-145 cells were treated with IL-4 to induce STAT6 phosphorylation with and without co-administration of cisplatin (10 μg/ml), oxaliplatin (14 or 25 μg/ml), or carboplatin (80 or 120 μg/ml) for 18 hours. Shown is one representative experiment performed in triplicate (+SEM) out of at least three independent experiments. (**C**) BLM (melanoma), DU-145 (prostate cancer), KATO-3 (gastric cancer), HT-29 (colon cancer) and HeLa (cervical cancer of the uterus) cells were treated with IL-4 or IL-6/IFNγ to induce STAT6 or STAT1 and 3 protein phosphorylation, respectively, with and without simultaneous co-administration of cisplatin (10 μg/ml; BLM cells 20 μg/ml) for 18 hours. STAT protein expression and phosphorylation was analyzed by western blot. Shown is one representative experiment out of at least 3 independent experiments. (**D**) DU-145 cells were treated with cytokines: IL-6 and IFNγ (STAT1 and STAT3) with and without co-administration of cisplatin (10 μg/ml) for 18 hours and STAT protein localization was visualized by confocal microscopy. Shown is one representative experiment out 3 independent experiments. (**E**) DU-145 cells were treated with IL-4 to induce STAT6 protein phosphorylation with and without co-administration of cisplatin (10 μg/ml), methotrexate (250 μg/ml), gemcitabine (20 μg/ml), cyclophosphamide (140 μg/ml) or vincristine (0.4 μg/ml) for 18 hours. STAT6 protein expression and phosphorylation were analyzed by western blot. Shown is one representative experiment out of 2 independent experiments. (**F**) DU-145 cells were pretreated or not with cisplatin for 12 hours and pulsed with a cytokine mix (IL-1β, TNF-α, IFNγ) for 30 minutes. ERK phosphorylation was analyzed by flow cytometry. Shown is one representative FACS plot and one representative experiment performed in triplicate (+SEM) out of 2 independent experiments. (**G**) DU-145 cells were treated with IL-4 to induce STAT6 protein phosphorylation with and without co-administration of cisplatin (10 μg/ml) for 18 hours. Akt protein expression and phosphorylation was analyzed by western blot. Shown is one representative experiment out of 2 independent experiments.

Having observed inhibition of STAT6 phosphorylation by platinum compounds [5], we hypothesized that other STATs might be affected as well. We chose to investigate this in DU-145 prostate cancer-derived cells, because they functionally express all STATs, except STAT4, which is predominantly expressed in lymphocytes. These STAT proteins, except STAT3, were observed in their inactive, unphosphorylated state, and could be activated by addition of appropriate cytokines (Figure 1A). There was a low level of STAT3 tyrosine phosphorylation, as previously reported for this cell line [10], likely induced by autocrine production of IL-6 which could be further increased by the addition of exogenous IL-6 (Figure 1A). Treatment with cisplatin induced dephosphorylation of all STATs as shown by western blot analysis (Figure 1A). Loss of STAT tyrosine phosphorylation was observed at clinically relevant concentrations of cisplatin, oxaliplatin and carboplatin (Figure 1B and Supplementary Figure 1A and 1B). To address the robustness of these findings we repeated these analyses in five tumor cell lines of distinct origin (melanoma, prostate, cervical, gastric, and colon cancer cell lines; Figure 1A and C), and found inhibited STAT phosphorylation in all cell lines. The inhibition of STAT phosphorylation coincided with reduced translocation of STAT proteins from the cytoplasm to the nucleus (Figure 1D). Other frequently used cytotoxic agents did not decrease STAT tyrosine phosphorylation levels (Figure 1E). In order to assess whether the observed platinum-associated inhibition of phosphorylation was specific for STAT proteins, we investigated the effect of cisplatin on ERK and Akt signaling. Consistent with previous reports [11], cisplatin did not inhibit ERK or Akt phosphorylation (Figure 1F and 1G), suggesting some specificity for interference with STAT proteins.

### Cisplatin inhibits de novo STAT protein phosphorylation

**Figure 2:**
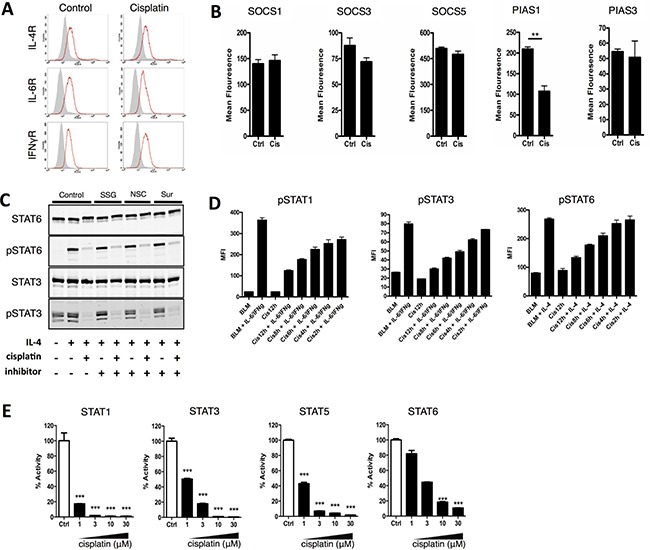
Cisplatin prevents STAT phosphorylation by binding to STAT proteins and blocking the SH2 domain (**A**) DU-145 cells were cultured with and without co-administration of cisplatin (10 μg/ml) for 24 hours and the cell surface expression of the IL-4, IFNγ and IL-6 receptor was determined by flow cytometry. Shown is one representative experiment out of two independent experiments. (**B**) DU-145 cells were cultured with and without co-administration of cisplatin (10 μg/ml) for 24 hours and the expression of SOCS1, SOCS2, SOCS3, PIAS1 and PIAS3 was determined by flow cytometry. Shown is one representative experiment performed in triplicate (+SEM) out of 2 independent experiments. (**C**) DU-145 cells cultured with IL-4 and with and without simultaneous co-administration of cisplatin (10 μg/ml) cisplatin in the presence of Sodium Stibogluconate (50 μg/ml), NSC87887 (50 μM) or Suramin (100 μg/ml). Phosphorylation of STAT6, 3 and 1 was analyzed by western blot. Shown is one representative experiment out of two independent experiments. (**D**) BLM cells were treated with cisplatin (20 μg/ml) for the indicated times after which the cells were stimulated with IL-6 and IFNγ (STAT1 and STAT3) or IL-4 (STAT6) for 30 minutes. STAT phosphorylation was measured by flow cytometry. Shown is the mean ± SEM of the mean fluorescence of one representative experiment out of at least 2 independent flow cytometry experiments performed in triplicate. (**E**) Binding of pTyr peptide to STAT SH2 domain visualized by normalized energy transfer between donor bead-coupled STATs and acceptor bead-coupled pTyr peptide (alpha screen). STATs were preincubated with increasing concentration of cisplatin (1, 3, 10 or 30 μM). Shown is the mean ± SEM of one representative experiment performed in triplicate out of 3 independent experiments.

To unravel the mechanism of action, we investigated whether the observed reduced level of STAT phosphorylation could be caused by downregulation of cytokine receptors. However, after incubating DU-145 cells with cisplatin we observed no effect on the cell surface expression of IL-4, IL-6 or IFNγ receptors (Figure 2A). Next, we focused on the main STAT deactivation pathways: (i) inactivation by suppressor of cytokine signaling (SOCS) proteins [12] or (ii) protein inhibitor of STAT (PIAS) proteins [13] or (iii) through dephosphorylation by SH2 domain-containing phosphatase (SHP) 1 and 2 [14]. The expression levels of these SOCS proteins, or of PIAS1 and PIAS3, did not increase after cisplatin treatment (Figure 2B and 2C). Finally, because a previous report showed that cisplatin could decrease the activity of JAK2 kinase by enhancing Shp-1 activity [15], we tested NSC-87877, suramin, or sodium stibogluconate, known inhibitors of phosphatases SHP 1 and 2, in combination with cisplatin, but found no effect on cisplatin-mediated downmodulation of STAT phosphorylation (Figure 2C). Taken together, these data indicate that the observed STAT dephosphorylation after platinum treatment was not caused by activation of STAT inhibitory pathways.

### Cisplatin directly binds STAT proteins and blocks the SH2 domain

We therefore focused on an often-neglected characteristic of platinum compounds, their ability to bind a variety of proteins [16]. We hypothesized that inhibition of STAT function might be attributed to a direct binding of cisplatin to STAT proteins. Tumor cells were pretreated with cisplatin for different time periods, washed and subsequently pulsed with cytokines after which STAT1, STAT3 or STAT6 phosphorylation was assessed. We observed that increased exposure to cisplatin correlated with reduced phosphorylation of STAT proteins (Figure 2D), indicating that cisplatin might inhibit *de novo* phosphorylation.

Next, we tested platinum drugs in an Alphascreen-based assay previously used to identify inhibitors that bind to the STAT3 SH2 domain [17]. This assay measures the binding of recombinant STAT proteins to a labeled phospho-tyrosine (pTyr) peptide, which corresponds with STAT docking sites of their corresponding upstream receptors (Supplementary Figure 2A and 2B). A non-labeled pTyr control peptide was used as a competitor and effectively blocked the binding of the FITC-labeled pTyr to STAT3 SH2 domain (Supplementary Figure 2C). While cisplatin by itself had no effect in the Alphascreen assay (Supplementary Figure 2D), incubation of STAT1 or STAT3 with cisplatin strongly inhibited binding of pTyr to the SH2 domain in a concentration dependent manner (Figure 2E). Blocking of the STAT SH2 domain by cisplatin was independent of the timing of addition *i.e*. before, simultaneous, or after addition of the pTyr (Supplementary Figure 2E). Inhibition of pTyr-SH2 domain interaction by cisplatin was also confirmed for STAT6 and STAT5b (Figure 2E). Similarly, another platinum compound frequently used in the clinic, oxaliplatin, also blocked pTyr-SH2 domain interaction (Supplementary Figure 3), although less potent than cisplatin. This may be caused by incomplete metabolization in the assay conditions as the active metabolite of oxaliplatin, DACH-platin, was as potent as cisplatin (Supplementary Figure 3). Indeed, DACH-platin also blocked STAT1, STAT5b and STAT6 SH2 domain binding (Supplementary Figure 4). This is in line with finding from others showing STAT3 SH2 domain targeting by preclinical non-platinum metal compounds [18, 19]. Collectively, these results suggest that platinum compounds target STAT molecules and block SH2 domain interactions. This direct binding prevents recruitment of STAT proteins to the receptor, thus inhibiting *de novo* STAT phosphorylation and dimerization and resulting in loss of (constitutive) STAT phosphorylation.

**Figure 3:**
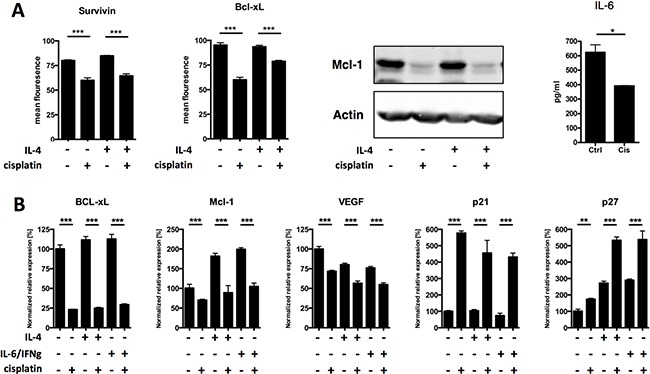
Platinum drugs downregulate STAT3 target genes (**A**) DU-145 cells were mock-treated or treated with cisplatin (with and without IL-4 or IL-6/IFNγ) and expression of STAT3 and STAT6 target genes survivin, Bcl-xL, Mcl-1, IL-6, VEGF was analyzed by flow cytometry (surviving, Bcl-xL), western blot (Mcl-1), or cytokine bead array (IL-6). (**B**) DU-145 cells were mock-treated or treated with cisplatin (with and without IL-4 or IL-6/IFNγ) and expression of STAT3 and STAT6 target genes survivin, Bcl-xL, Mcl-1, IL-6, VEGF were analyzed by quantitative RT-PCR, together with expression of p53 target genes p21 and p27. Shown is one representative experiment performed in triplicate (+SEM) out of at least three independent experiments.

To investigate the functional consequences of platinum-induced loss of STAT phosphorylation on downstream effector pathways, we focused on the expression of STAT3 and STAT6 target genes that have known oncogenic effects, BCL-X_L_, MCL-1, survivin and VEGF. We found that cisplatin treatment caused a significant downregulation of these genes, both at the protein (Figure 3A) and the transcriptional (Figure 3B) level. Furthermore, production of the STAT3-driven cytokine IL-6 was also inhibited (Figure 3A). In contrast, p53-driven upregulation of cell cycle inhibitors p21 and p27 was unaffected (Figure 3B), indicating that these cells were transcriptionally active and that cisplatin specifically downregulated the expression of STAT target genes. Together these data demonstrate that platinum-induced STAT dephosphorylation results in the inhibition of downstream pro-survival and anti-apoptotic target genes.

### STAT protein activity in tumor cells predicts outcome to platinum-based therapy

**Figure 4:**
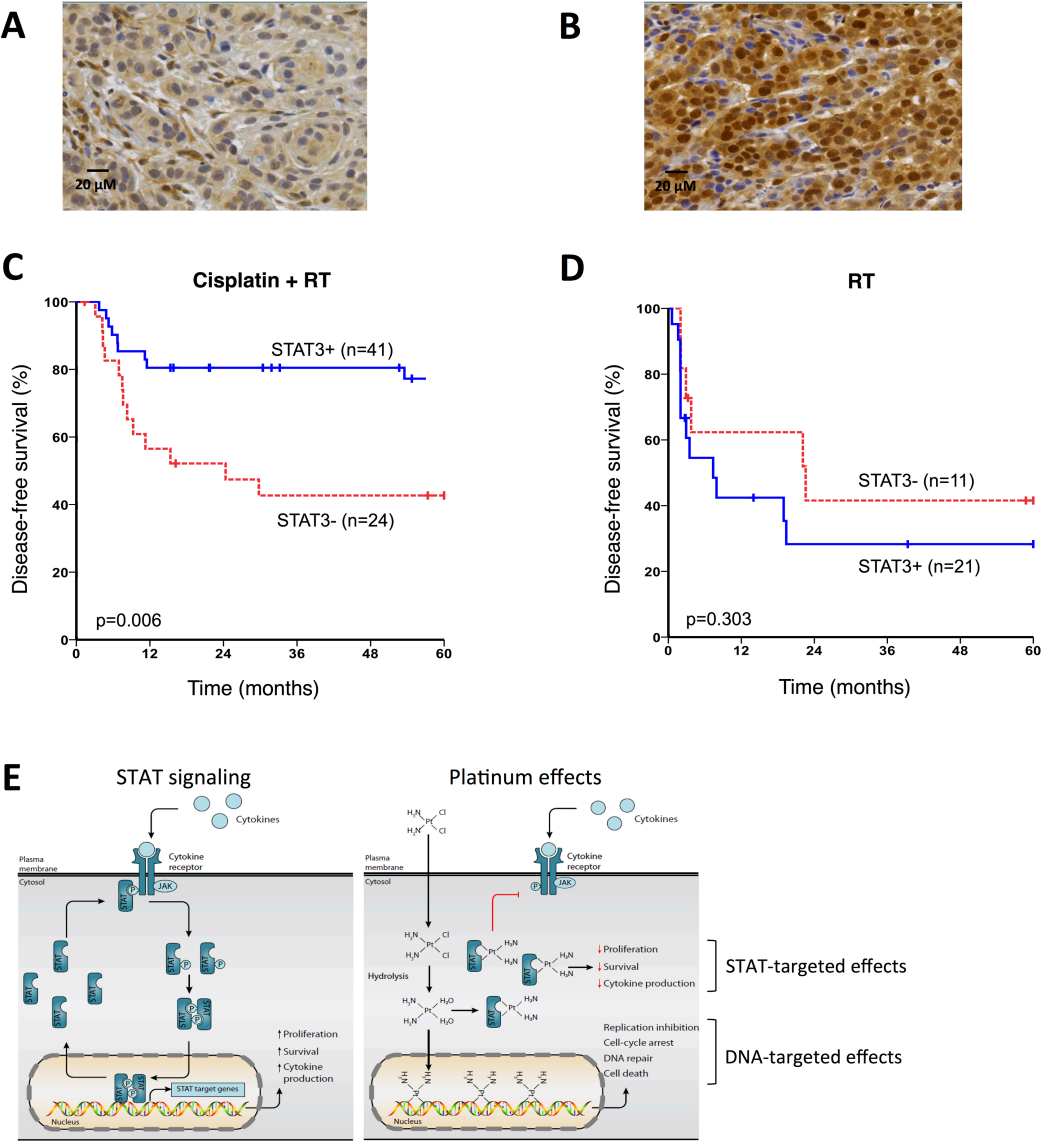
Activated STAT3 in the tumour highly significantly correlates with improved survival after platinum-based therapy in patients (**A**) Example STAT3 staining of HNSCC tumor showing predominantly cytoplasmic STAT3 localization. (**B**) Example STAT3 staining of HNSCC tumor showing predominantly nuclear STAT3 localization. (**C**) Kaplan-Meier estimates of disease-free survival (*p* = 0.006) of head and neck cancer patients with (*n* = 41) or without (*n* = 24) tumor nuclear STAT3 expression that had been treated with cisplatin and radiotherapy. (**D**) Kaplan-Meier estimates of disease-free survival (*p* = 0.303) of head and neck cancer patients with (*n* = 21) or without (*n* = 11) tumor nuclear STAT3 expression that had been treated with radiotherapy alone. (**E**) General STAT signaling pathway and proposed mechanism of action for platinum compounds.

To investigate the clinical relevance of platinum-induced STAT protein modulation in tumor cells in cancer patients, we analyzed expression of STAT3 in tumors of two cohorts of patients with head and neck squamous cell carcinoma (HNSCC) who had been treated with either cisplatin-based chemo-radiotherapy (*n* = 65) or radiotherapy alone (*n* = 32). We chose this patient category because it allowed us to assess the influence of STAT3 on cisplatin monotherapy efficacy. We discriminated between inactive STAT3 (cytoplasmic localization; STAT3 negative; Figure 4A) and active STAT3 (nuclear localization; STAT3 positive; Figure 4B). After a maximum follow-up of 60 months, we observed that the presence of active STAT3 was highly significantly correlated with improved disease-free survival (DFS, *p* = 0.006) after platinum-based chemo-radiotherapy. Five-year DFS rates was 77.3% for patients with STAT3-positive tumors versus only 42.7% for patients with STAT3-negative tumors (Figure 4C), even though patients with STAT3 positive tumors had a more advanced tumor stage in our cohort (Supplementary Table 1). Importantly, this correlation between tumor STAT3 expression and response to treatment was not present in patients treated with radiotherapy alone (Figure 4D), indicating that the observed correlation is specific for the platinum therapy. In addition, this correlation was only present for tumor cells positive for nuclear STAT3, not for lymphocytic infiltrates (*p* 0.788), suggesting that the clinical effect of STAT modulation predominantly plays a role via the tumor cells themselves.

## DISCUSSION

Considering the importance of STAT signaling during cancer development, many studies explored the inhibition of STAT signaling as a cancer intervention strategy [8]. We demonstrate that STAT3 and other STATs are direct targets of platinum compounds, the most commonly used group of chemotherapeutics. Platinum compounds bind to STAT proteins, thereby blocking their SH2 domain thus preventing tyrosine phosphorylation and nuclear translocation of these transcription factors, ultimately resulting in downregulation of tumor-promoting STAT target genes (Figure 4E). Novel experimental platinum compounds were reported to impair STAT3 function, mainly by interfering with STAT3 DNA binding activity, leading the authors to propose their use in the clinic as STAT3 inhibitors [6, 7]. We found that the STAT signaling inhibiting potential of clinically used platinum compounds cisplatin, carboplatin and oxaliplatin was caused by a direct binding of STAT proteins rather than through DNA binding. The robustness of our findings is underpinned by the reproducibility in multiple cell lines, using multiple platinum compounds at clinically relevant concentrations. In addition, recent *in vitro* findings using experimental, non-platinum, metal compounds that target STAT3 SH2 domain, further substantiate the notion that platinum compounds directly bind to STAT proteins [18, 19]. However, the exact binding site of platinum compounds remains obscure.

STAT3, STAT5, and STAT6 exhibit tumor-promoting functions, not only in cancer cells and cancer stem cells, but also in other cellular constituents of the tumor microenvironment, such as stromal cells and immune cells [9, 20]. As we previously showed that the ability of platinum drugs to modulate STAT signaling was not restricted to cancer cells but was also evident in immune cells [5], we postulate that these compounds have the ability to modulate the entire tumor microenvironment, specifically by antagonizing immune evasive and suppressive networks. These include, secretion of inhibitory and inflammatory cytokines and expression of immune checkpoints, such as PD-L1 and PD-L2 [5, 21, 22]. However, our data here show that these drugs also bind STATs that are associated with Th1 immune responses and therefore may contribute to anti-tumor immunity, such as STAT1 [20]. We hypothesize that the ultimate outcome of platinum induced STAT inhibition may be dependent on a delicate balance between inhibition of tumorigenic STATs and tumor antagonizing STATs, which is further dependent on which STATs are expressed by the tumor cells and infiltrating immune cells.

Biomarkers that can predict response to therapy are available for several oncogene-targeted therapies, immunotherapies and hormonal therapies, but to date there is no validated biomarker that is able to predict response to cytotoxic chemotherapy. Given the very substantial toxicity associated with these drugs, balanced against sometimes modest response rates, there is a strong need for predictive biomarkers. As an example, the addition of cisplatin treatment to radiotherapy for HNSCC patients results in a modest increase in 5-year PFS from 27.2% to 33.7% [23]. This means that for the vast majority of patients, the addition of cisplatin is not beneficial. In our analysis, tumor cell nuclear STAT3 expression directly correlated with an improved disease-free and overall survival in patients treated with cisplatin-based chemoradiotherapy, while there was no such correlation for patients treated with radiotherapy alone. Although it is tempting to speculate that patients with STAT3 negative tumors did not benefit from the addition of cisplatin since the STAT3 negative arms displayed a comparable DFS for patients treated with or without cisplatin, the retrospective and non-randomized nature of our study does not allow such a conclusion. However, if confirmed, STAT3 may serve as a predictive biomarker that can be used to predict whether a patient is likely or not to benefit from platinum-based therapy.

Taken together, our data provide novel insights into the complex biology underlying the clinical efficacy of platinum compounds. These results may lead to the development of innovative treatment schedules, exploiting this novel STAT inhibiting effect of platinum-based chemotherapy to potentiate other therapeutic strategies such as immunotherapy or oncogene-targeted therapies, and have the potential to provide first possibility to personalize chemotherapy treatment in cancer.

## MATERIALS AND METHODS

### Cell culture

BLM (described in [5]) cells were cultured in DMEM (5% FCS), Hela (described in [24]) cells in DMEM (10% FCS). DU-145 and HT-29 (obtained from Dr. Wieger Norde, Radboud UMC) cells were cultured in RPMI (10% FCS). KATO-3 (obtained from Dr. Hanneke van Laarhoven, Radboud UMC) cells were cultured in IMDM (20% FCS). Cell lines were not authenticated. Where indicated cytokines were added to culture medium: IL-4 (300 U/ml), IFNγ (400 U/ml) or IL-6 (15 ng/ml) (all Cellgenix).

### Preparation of protein lysates and Western blotting

Cells were washed with PBS and harvested using TEN harvest buffer (10 mM Tris pH7.8, 5 mM EDTA, 50 mM NaCl). Cell pellets were lysed in TEN lysis buffer (TEN, 1% Triton X-100, 1 mM PMSF, 10 μg/ml aprotinin, 10 μg/ml leupeptin, 1 mM sodium orthovanadate, 10 mM pyrophosphate, 50 mM NaF, 1X Roche protease inhibitor cocktail (Roche Diagnostics)) on ice. Equal amounts of protein were denatured in Laemmli sample buffer and subjected to SDS-PAGE and Western blotting. After blocking, membranes were incubated with primary anti-STAT antibodies, washed again and subsequently incubated with polyclonal goat anti-rabbit Alexa Fluor-680 (Molecular Probes, Eugene, OR) and goat anti-mouse IRDye800CW (LI-COR Biosciences) as a secondary antibody, and analyzed with the LICOR Odyssey Imaging system (LI- COR Biosciences).

### Native PAGE

DU-145 cells were treated with IL-4 to induce phosphorylation and dimerization of STAT6, in presence or absence of cisplatin. Harvesting of cells, preparation of cell lysates is described above; native page was performed as described previously [25].

### Cell surface staining of cytokine receptors

Cells were harvested using TEN harvest buffer, washed with PBS, 1% albumin, 0,05% Sodium Azide (PBA) and incubated with primary antibody (mouse anti-human CD119, mouse anti-human CD124 or mouse anti-human CD126; all BD Pharmingen), washed with PBA and incubated with biotinylated goat anti-mouse IgG1 (BD Pharmingen). Cells were washed with PBA and incubated with PE-Streptavidin (BD Pharmingen), washed and analyzed on a FACScalibur.

### Intracellular staining for STAT proteins

Cells were harvested and fixed with 4% formaldehyde by 10 min incubation at 37°C. Cells were permeabilized by addition of ice cold 90% methanol and incubated on ice for 30 min. Permeabilized cells were washed with PBA and incubated with primary antibody, washed twice with PBA and incubated with secondary antibody (Goat anti-mouse- alexa647 or Goat anti-rabbit-alexa647 were used as secondary antibodies), washed again and analyzed on a FACScalibur.

### Intracellular STAT protein localization by confocal microscopy

Cells were adhered to 12 mm coverslips and stimulated with cytokines to induce STAT phosphorylation and translocation and treated with or without cisplatin (10 μg/mL, Pharmachemie BV). After 18 hours cells were fixed with 4% PFA and subsequently quenched with CLSM buffer (PBS, 3% BSA, 10 mM glycine). Cell membranes were stained with PKH-26 (2 × 10^–3^ mM, Sigma Aldrich) and reaction was quenched by addition of FCS. Cells were permeabilized with 96% methanol (–20°C) for 5 minutes at 4°C, quenched with CLSM buffer and incubated with primary antibody. After washing, cells were stained with an Alexa-647 conjugated secondary antibody. Nuclei of the cells were stained with DAPI and cover slips were sealed on microscope plates in Mowiol (Calbiochem). Analysis was done with an Olympus FV1000 Confocal Laser Scanning Microscope.

### Antibodies

Mouse monoclonal anti-β-actin (Sigma-Aldrich), mouse monoclonal anti-pSTAT6(Tyr641) (BD Biosciences Pharmigen), rabbit polyclonal anti-STAT6 antibody (Santa Cruz Biotechnology). Rabbit polyclonals anti-pERK1/2-alexa647, anti-Akt and anti-pAkt(S473), anti-STAT1, anti-pSTAT1(Tyr701), anti-STAT2, anti-pSTAT2(Tyr690), anti-STAT3, anti-pSTAT3(Tyr705), anti-STAT5, anti-pSTAT5(Tyr694), anti-pSTAT1(Ser727) and anti-pSTAT3(Ser727) were all from Cell Signaling. Anti-STAT1, anti-pSTAT1(Tyr701), anti-STAT3, anti-pSTAT3(Tyr705) antibodies recognize both the a-isoform and the b-isoform of the respective STAT protein.

### Realtime quantitative reverse transcription PCR

Cells were treated as mentioned and after 18 hours total RNA was extracted using Zymo Research Quick-RNA^TM^ MiniPrep kit according to the manufacturer's instructions. RNA was treated with DNase-1 prior to reverse transcription. Real-time Quantitative PCR was performed using Power Sybr^®^Green (Applied Biosciences) on a Bio-Rad CFX 96 RealTime System C100 Thermal Cycler. Relative gene expression levels were determined by normalization to β-actin level using the ΔΔCt method. Primers sequences are available upon request.

### STAT Alphascreen

Recombinant human STAT proteins were expressed and subsequently biotinylated through avi-tag introduced at the N-terminus of the proteins. In brief, biotinylated STAT was incubated for 90 min with a platinum compound and FITC-pTyr peptide, and mixed with streptavidin-coated donor beads and anti-FITC acceptor beads simultaneously before detection at 570 nm using EnVison Xcite (PerkinElmer). Phospho-Tyr (pTyr) peptide probes used in this study were 5-carboxyfluorescein (FITC)-GpYLPQTV (STAT3), FITC- GpYDKPHVL (STAT1), FITC-GpYKPFQDL (STAT6), FITC-GpYLVLDKW (STAT5b). For STAT6 and STAT5 a truncated form of the protein was used in which the N-terminal domain of both of these proteins was truncated. The full-length proteins could not be purified in a functional form. A control experiment using a truncated STAT3 protein (ΔN-STAT3) showed that the truncated protein behaved the same as the full-length protein in the Alphascreen and was similarly inhibited by cisplatin (Supplementary Figure 3).

### Statistical analysis

Biological data was analyzed by student's t test or one-way anova. *p < 0.05, **p < 0.01, ***p < 0.001. DFS and OS curves were estimated by the Kaplan-Meier method and compared by means of the log-rank test using IBM SPSS Statistics (SPSS version 20.0) software (SPSS Inc, Chicago, IL).

### Clinical study

We retrospectively analyzed data from 2 patient cohorts: one cohort of patients that had been treated with weekly cisplatin 40 mg/m2 in combination with a 6-week course of radiotherapy and a second cohort of patients that had been treated with radiotherapy alone in our institute in the period of 2000–2010. Eligible patients had histologically proven locally advanced squamous cell carcinoma of the head and neck without distant metastases and histological tumor material for STAT staining obtained before treatment had to be available. These patients were informed that their clinical data and tissue samples could be used for anonymized scientific analysis, for which they gave their consent. The following characteristics were registered in the database: tumor location, TNM stage, age, and sex. We collected data of 65 patients that had been treated with cisplatin in combination with radiotherapy and of 32 patients who had been treated with radiotherapy alone.

### Immunohistochemical scoring

All specimens were scored independently by two clinical pathologists (JvK and AH), who were blinded for clinical parameters or treatment outcomes. In all discrepant cases between the two observers (25% for STAT3), consensus was reached after discussion at a two-headed microscope. Inflammatory cells that stain for STAT3 were used as internal control. Scoring of STAT3 positive nuclear or cytoplasmatic staining was performed using the following categories: 0–10, 11–20, 21–30, 31–40, 41–50, 51–60, 61–70, 71–80, 81–90 and 91–100%. Based on the distribution, 50% positive cells was chosen as cut-off level: tumors were considered positive, when more than 50% of the cells had either cytoplasmatic or nuclear staining. Furthermore, the pattern of staining was scored as either being mostly nuclear or cytoplasmatic.

### IHC staining procedure for STAT3

Slides of 4-μm thickness were cut from formalin-fixed, paraffin-embedded (FFPE) tissue blocks. Subsequently, slides were deparaffinized in xylene for 5 min followed by rehydration in 100% ethanol and tapwater. Next, antigen retrieval using 10 mM citrate buffer (pH 6.0; Skytek, Logan, UT) was performed for 10 min at 96°C using the PT-module (Thermo scientific, labvision). After rinsing in PBS, slides were placed in an Autostainer 480 (Thermo Scientific, Waltham, MA). In the stainer, the endogenous peroxidase was blocked using 3% hydrogen peroxidase in methanol (both EMD Millipore corporation, Darmstadt, Germany) followed by primary antibody incubation, STAT3(Cell Signaling, dilution: 1/100) for 60 min at room temperature. The secondary antibody was Brightvision poly-HRP-anti Ms/Rb/Rt IgG (Immunologic BV, Duiven, Netherlands, Dilution: 1/2) which was incubated for 30 minutes at room temperature, followed by a visualization step with DAB (Bright-DAB Substrate Kit, Immunologic, Duiven, Netherlands) for 7 min at RT. After visualization, slides were counterstained with haematoxylin and mounted with Quick-D mounting medium (Klinipath, Duiven, Netherlands).

## SUPPLEMENTARY MATERIALS FIGURES AND TABLE


