# Supplementary material for: Direct inhibition of STAT signaling by platinum drugs contributes to their anti-cancer activity
Source: Oncotarget. 2017 May 7;8(33):54434–43. doi: 10.18632/oncotarget.17661 (PMC5589592; doi:10.18632/oncotarget.17661)
Supplement: Supplementary file 1 [file oncotarget-08-54434-s001.pdf]

Direct inhibition of STAT signaling by platinum drugs contributes to their anti-cancer activity

Supplementary Materials

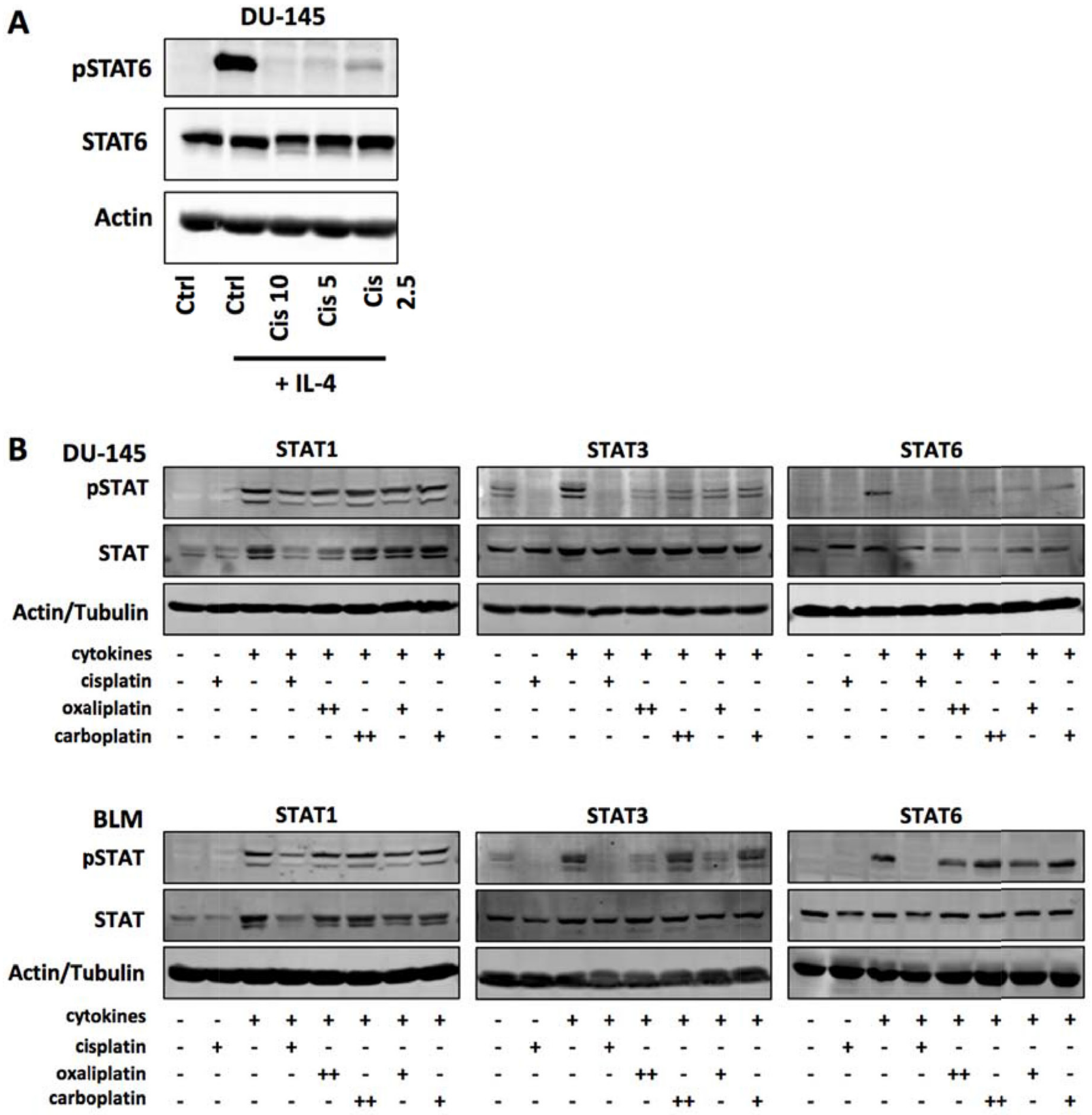

Supplementary Figure 1: (**A**) DU-145 cells were treated with IL-4 to induce STAT6 protein phosphorylation with and without co-administration of cisplatin (10, 5 or 2.5 µg/ml) for 18 hours; maximum concentration found in blood is approx. 5 µg/ml. STAT6 expression and phosphorylation was analyzed by western blot. Shown is one representative experiment out of 2 independent experiments. (**B**) DU-145 or BLM cells were treated with IL-6/IFNγ to induce STAT1 and STAT3 phosphorylation or IL-4 to induce STAT6 phosphorylation with and without co-administration of cisplatin (10 µg/ml), oxaliplatin (25 or 14 µg/ml), or carboplatin (120 or 80 µg/ml) for 18 hours. Tubulin (pSTAT1 and pSTAT3) or Actin (pSTAT6) is shown as a loading control. Shown are two independent experiments.

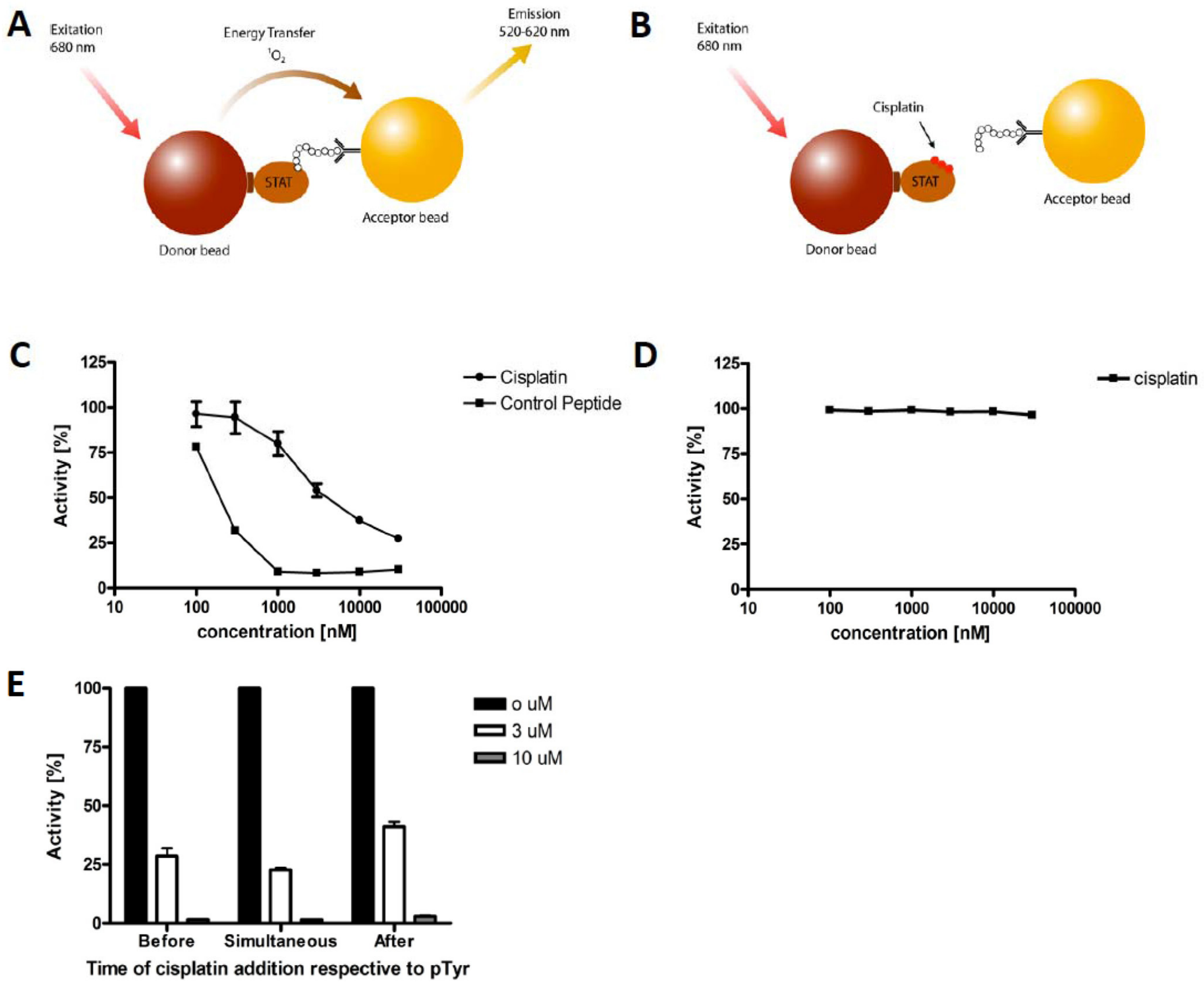

Supplementary Figure 2: (**A**) Recombinant STAT protein is biotynilated and pTyr peptide is coupled to FITC. pTyr binds to the STAT SH2 domain. Streptavidin-coupled donor beads and anti-FITC antibody-coupled acceptor beads are added to the mix. Binding of pTyr to STAT SH2 domain brings donor- and acceptor beads in close proximity, thereby allowing energy transfer via singlet oxygen molecules leading to fluorescence emission by the acceptor beads. (**B**) Hypothesis: cisplatin binds to and blocks the STAT SH2 domain thus inhibiting pTyr binding and no energy is transferred to the acceptor beads. (**C**) A non-labeled pTyr peptide was added to the assay as a competitor. (**D**) Biotin donor beads were incubated with streptadivin-coupled acceptor beads in the presence of cisplatin and the fluorescence was measured. (**E**) Cisplatin was added to donor bead-coupled STAT3 15 min before or at the same time as addition of acceptor bead-coupled pTyr peptide. Emission was measured 90 minutes after addition of pTyr. For the after samples: STAT3 was incubated with pTyr for 90 minutes and cisplatin was added before measurement of emission.

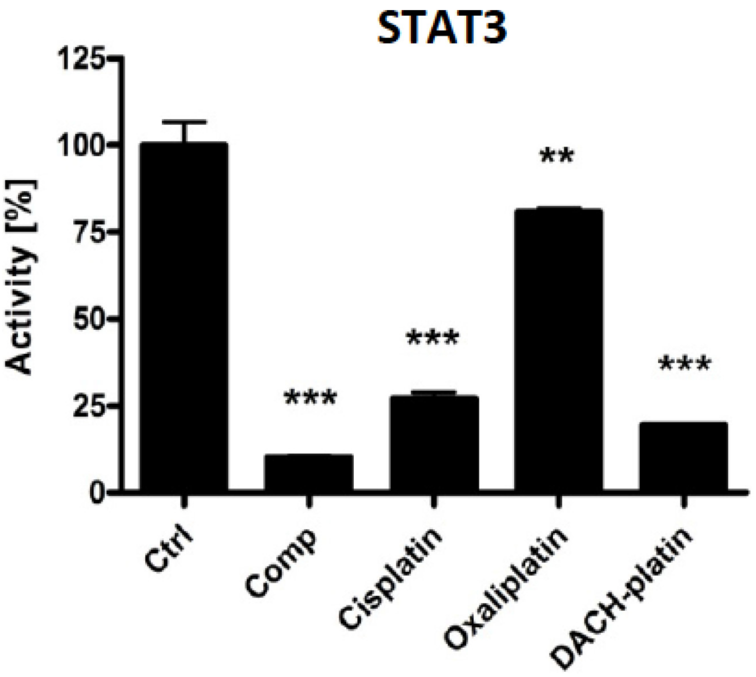

Supplementary Figure 3: The active metabolite of oxaliplatin, DACH-platin, was equally effective as cisplatin in blocking the STAT3 SH2 domain (all compounds used at 30 µM).

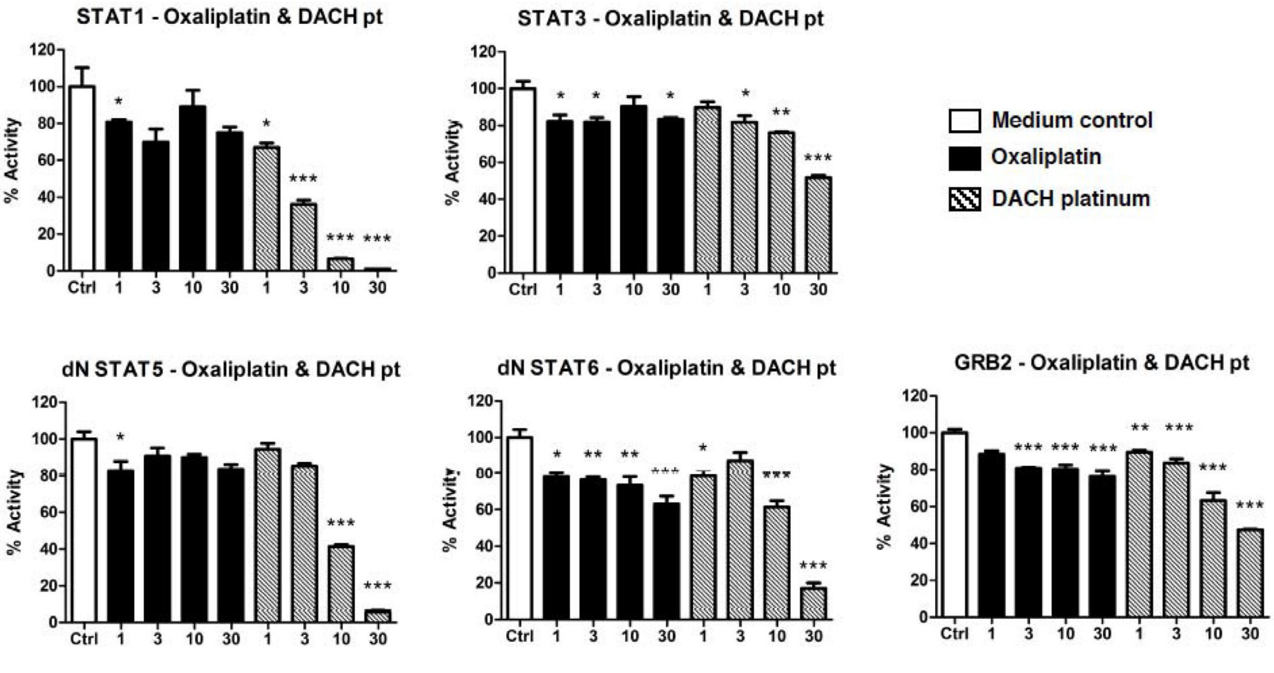

Supplementary Figure 4: Results of Alphascreen assay showing the ability of oxaliplatin and its metabolite DACH-platin to block the SH2 domains of STAT1, STAT3, STAT5, STAT6 and GRB2.

Supplementary Table 1: Patient characteristics

	**CIS + RT**		**RT**	
	**STAT3+ *n* = 41**	**STAT3− *n* = 24**	**STAT3+ *n* = 21**	**STAT3− *n* = 11**
**Age (median)**	59 (43–71)	56 (32–65)	60 (46–82)	61 (41–78)
**Localisation**		***p* = 0.311***		***p* = 0.269**
hypopharynx	18 (44%)	6 (25%)	9 (43%)	4 (36%)
oropharynx	17 (41%)	13 (54%)	6 (29%)	1 (9%)
other	6 (15%)	5 (21%)	6 (29%)	6 (55%)
**T stage**		***p* = 0.058**		***p* = 0.531**
T1	2 (5%)	0 (0%)	0 (0%)	0 (0%)
T2	8 (20%)	0 (0%)	2 (10%)	0 (0%)
T3	15 (37%)	14 (58%)	9 (43%)	6 (55%)
T4	16 (39%)	10 (42%)	10 (48%)	5 (45%)
**N stage**		***p* = 0.034**		***p* = 0.013**
N0	6 (15%)	1 (4%)	0 (0%)	1 (9%)
N1	2 (5%)	7 (29%)	4 (19%)	5 (45%)
N2	32 (78%)	15 (63%)	17 (81%)	3 (27%)
N3	1 (2%)	1 (4%)	0 (0%)	2 (18%)

**p* with chi-square.
